# Novel *β*-carbolines against colorectal cancer cell growth via inhibition of Wnt/*β*-catenin signaling

**DOI:** 10.1038/cddiscovery.2015.33

**Published:** 2015-10-05

**Authors:** X Li, B Bai, L Liu, P Ma, L Kong, J Yan, J Zhang, Z Ye, H Zhou, B Mao, H Zhu, Y Li

**Affiliations:** 1 State Key Laboratory of Phytochemistry and Plant Resources in West China, Kunming Institute of Botany, Chinese Academy of Sciences, Kunming 650201, People’s Republic of China; 2 School of Food and Biological Engineering, Zhengzhou University of Light Industry, Zhengzhou 450002, People’s Republic of China; 3 Chinese Center for Chirality, Key Laboratory of Medicinal Chemistry and Molecular Diagnostics of Education Committee of China, Hebei University, Baoding 071002, People’s Republic of China; 4 State Key Laboratory of Genetic Resources and Evolution, Kunming Institute of Zoology, Chinese Academy of Sciences, Kunming 650201, People’s Republic of China

## Abstract

Wnt signaling pathway is aberrantly activated in a variety of cancers, especially in colorectal cancer (CRC), because of mutations in the genes encoding adenomatous polyposis coli (APC), *β*-catenin and Axin. Small-molecule antagonists of Wnt/*β*-catenin signaling are attractive candidates for developing effective therapeutics for CRC. In this study, we have identified a novel Wnt signaling inhibitor, isopropyl 9-ethyl-1- (naphthalen-1-yl)-9H-pyrido[3,4-b]indole-3- carboxylate (Z86). Z86 inhibited Wnt reporter activities and the expression of endogenous Wnt signaling target genes in mammalian cells and antagonized the second axis formation of *Xenopus* embryos induced by Wnt8. We showed that Z86 treatment inhibits GSK3β (Ser9) phosphorylation, leading to its overactivation and promoting the phosphorylation and degradation of *β*-catenin. *In vitro*, Z86 selectively inhibited the growth of CRC cells with constitutive Wnt signaling and caused obvious G1-phase arrest of the cell cycle. Notably, in a nude mouse model, Z86 inhibited dramatically the xenografted tumor growth of CRC. Daily intraperitoneal injection of Z86 at 5 mg/kg resulted in >70% reduction in the tumor weight of HCT116 cell origin that was associated with decreased GSK3*β* (Ser9) phosphorylation and increased *β*-catenin phosphorylation. Taken together, our findings provide a novel promising chemotype for CRC therapeutics development targeting the canonical Wnt signaling.

## Introduction

The Wnt signaling transduction pathway plays a critical role in embryonic developmental processes, tissue homeostasis and tumorigensis.^1^ *β*-Catenin is an important component of the canonical Wnt signaling pathway. In the absence of Wnt ligands, *β*-catenin is phosphorylated by a destruction complex including adenomatous polyposis coli (APC)/Dsh/Axin/GSK3*β* that is then ubiquitinated and degraded through the proteasome pathway, keeping *β*-catenin at a low level in the cytoplasm. Once Wnt signaling pathway is activated, glycogen synthase kinase-3*β* (GSK3β) is inhibited and cytosolic *β*-catenin becomes stabilized. The accumulated *β*-catenin translocates to the nucleus and interacts with T-cell factor (TCF)/lymphoid enhancer factor (LEF) that activates transcription of Wnt target genes involved in cell proliferation and cell-fate determination.^2^ Numerous studies suggest that aberrant activation of the Wnt/*β*-catenin signaling pathway plays an important role in human tumorigenesis.^3^ In colon cancer, mutant APC and *β*-catenin highlighted the implications of aberrant Wnt/*β*-catenin signaling in the development and progression of this malignant phenotype.^4^ *β*-Catenin mutations and elevated levels of nuclear *β*-catenin, both hallmarks of active Wnt signaling, have also been reported in colorectal cancer (CRC).^5,6^ The abnormal activation of Wnt signaling increased the expression of Wnt target genes such as cyclin D1,^7,8^ c-MYC (v-myc avian myelocytomatosis viral oncogene homolog)^9^ and survivin^10^ that are involved in proliferation, apoptosis and cell cycle deregulation in many human malignancies. Accordingly, small-molecule antagonists of Wnt/*β*-catenin signaling have become attractive candidates for developing effective therapies for CRC.^11,12^

Based on assays of the transcriptional complexes, several small molecules were first identified as Wnt inhibitors that inhibited CRC cell proliferation and interfered with *β*-catenin-mediated axis duplication in *Xenopus* embryos.^13,14^ Through cell-based transcriptional reporter assay screening, additional Wnt inhibitors were identified that works through inhibiting the interaction between *β*-catenin and TCF/LEF transcriptional complexes in CRC.^15–18^ Recently, several small-molecule Wnt inhibitors targeting the destruction complex composed of APC, Axin, GSK3*β* and other proteins were discovered. Huang *et al.*^19^ found that small-molecule compound XAV939 promoted the phosphorylation and subsequent degradation of *β*-catenin by stabilizing of Axin, thus causing inhibition of Wnt signaling and colorectal tumorigenesis. Several other studies have also confirmed the effectiveness of suppression of APC mutation-driven colorectal tumor growth through inhibition of Wnt signaling pathway by stabilizing Axin.^20–22^

In the present study, we have identified a novel Wnt signaling inhibitor, isopropyl 9-ethyl-1-(naphthalen-1-yl)-9H-pyrido[3,4-b]-indole-3-carboxylate (Z86), that belongs to *β*-carboline structure-type compound. This type of compounds was first derived from natural alkaloids. In recent several decades, plenty of novel *β*-carboline-type compounds were designed and synthesized based on significant antitumor activity *in vitro*.^23,24^ Our study found that Z86 inhibited Wnt signaling through activation of GSK3*β*, leading to increased phosphorylation and degradation of *β*-catenin. Z86 reduced the secondary body axis formation induced by Wnt8 in *Xenopu*s embryos. *In vitro*, Z86 exhibited selective inhibitory effects on the proliferation of colon cancer cells, and dramatically suppressed tumor growth of CRC HCT116 xenografts *in vivo*, both of which were mediated by inhibition of Wnt signaling. The identified chemotype Z86 may serve as a promising start point for the development of novel CRC therapeutics and deserve further evaluation.

## Results

### Identification of Z86 as a potent antagonist of canonical Wnt signaling

**Figure 1:**
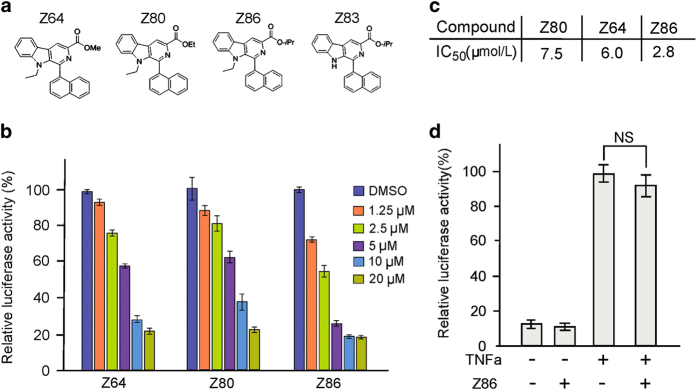
Identification of Z86 as a potent antagonist of Wnt signaling. (**a**) Structures of the *β*-carboline compounds. (**b**) Effects of Z64, Z80 and Z86 on the Topflash reporter activity. The HEK293W cells were treated with different doses of Z64, Z80 and Z86 respectively for 24 h. Relative luciferase activity (Topflash/*Renilla*) measured represents the level of activated Wnt signaling. (**c**) The calculated IC_50_ values of Wnt signaling inhibition of the compounds are displayed in the table. (**d**) Z86 preferentially inhibits Wnt signaling (ST-Luc) over the NF-*κ*B signaling pathway. NF-*κ*B signaling was stimulated with 25 ng/ml TNF*α* treatment for 24 h in HEK293T cells that were subsequently treated with Z86 (20 *μ*M) for 24 h and the NF-*κ*B-Luc luciferase activity was measured. Each bar is the mean±S.D. from three independent experiments. NS, not significant, relative to the vehicle control.

To identify compounds that inhibit the canonical Wnt signaling pathway, a chemical library consisting of 4000 chemically diverse compounds was screened. Our screening led to the identification of *β*-carboline structure type as a new class of inhibitors of Wnt signaling. Followed by this discovery, a library of *β*-carboline derivatives (∼150 compounds) were synthesized (Supplementary Figure S1). The inhibitory activities of the derivatives on Wnt signaling were investigated and the structure–activity relationship was characterized. As shown in Figures 1a–c, compounds Z64, Z80 and Z86, which bear a methyl, ethyl and isopropyl ester substituent at C-3 position and with substituents at indole N-9 position, exhibited strong activities to inhibit the canonical Wnt signaling in the SuperTopflash reporter activity assay with median inhibitory concentration (IC_50_) values of 6.0, 7.5 and 2.8 *μ*M, respectively. However, Z83 without substituents at indole N-9 position only exhibited weak activity with IC_50_ values of >20 *μ*M. These results suggested that the N-9 substituents of *β*-carboline played an important role in the Wnt inhibition activity. Furthermore, compounds with a bulky group possess a better activity than with a smaller group like methyl or ethyl ester substituent at C-3 position. To test the specificity of the inhibitory activity of Z86 on Wnt signaling, we performed nuclear factor (NF)-*κ*B reporter (NF-*κ*B-Luc) activity assays. As shown in Figure 1d, Z86 had little effects on the reporter activity of NF-*κ*B signaling at the concentration that significantly inhibited the SuperTopflash activity in HEK293T cells.

### Z86 inhibits Wnt signaling in colon cancer cells and blocks secondary axis formation in *Xenopus* embryos

**Figure 2:**
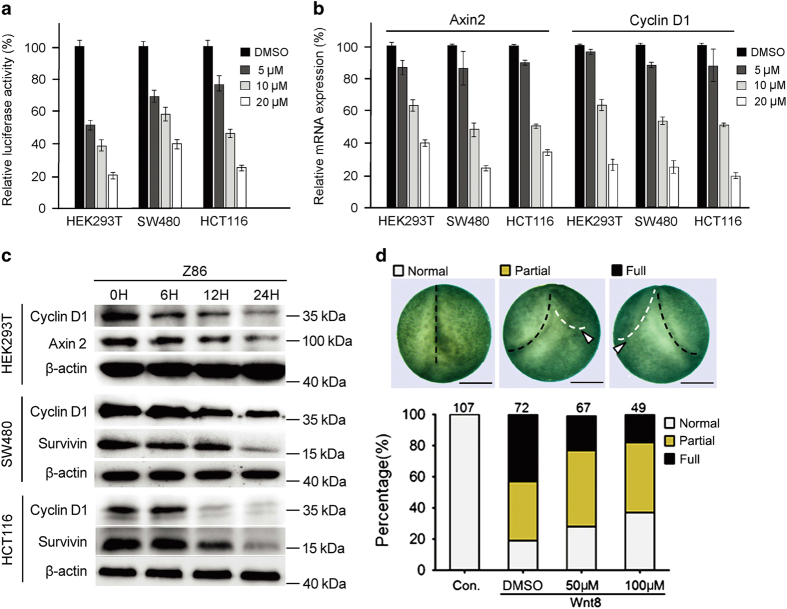
Z86 inhibits Wnt signaling in colon cancer cells and the secondary axis formation in *Xenopus* embryos. (**a**) Z86 inhibits Topflash reporter activity in a dose-dependent manner in *Wnt1*-transfected HEK293T cells and CRC cells, SW480 and HCT116. Cells were transfected with luciferase reporters (*Wnt1* was co-transfected into HEK293T cells) for 3 h, and subsequently were incubated with Z86 with indicated dosages for additional 24 h. Luciferase activity was then measured. (**b**) Z86 inhibits Axin2 and cyclin D1 expression in *Wnt1-*transfected HEK293T cells and CRC cells. The HEK293T cells were transfected with *Wnt1* 3 h before Z86 treatment. The cells were treated with indicated dosages of Z86 for 12 h. The levels of Axin2 and cyclin D1 mRNAs were determined by real-time PCR and normalized to *β*-actin. (**c**) Effects of Z86 on the protein expression of Axin2, Cyclin D1 and Survivin in *Wnt1-*transfected HEK293T cells and CRC cells. The HEK293T cells were transfected with *Wnt1* 3 h before Z86 treatment. The cells were treated with Z86 (20 *μ*M) for different time periods and cell extracts were harvested and assayed for the expressions of indicated proteins by western blotting. *β*-Actin was used as the loading control. (**d**) Z86 inhibits the secondary axis (white arrowheads) induced in *Xenopus* embryos by ventrally injected Wnt8 mRNA in a dose-dependent manner. The *Wnt8-*injected embryos were treated with DMSO or Z86 and were examined for axis duplication (white arrowhead in the upper panel) at late neurula stage (stage 18). Representative embryos are shown in the upper panel (dorsal views with anterior to the bottom). The statistic data are shown in the lower panel and the numbers of embryos in each group are shown on the top of each column. Scale bars=0.5 mm.

The inhibitory activity on Wnt signaling of Z86 was confirmed in *Wnt1* transiently transfected HEK293T cells (Figure 2a). Aberrant activation of Wnt signaling occurs in up to 80% of colon cancers. We further examined the effects of Z86 on endogenous Wnt signaling in colon cancer cell lines, SW480 (with APC deficiency) and HCT116 (harboring mutated *β*-catenin), in which the Wnt/*β*-catenin signaling pathway is constitutively activated. As in *Wnt1*-transfected HEK293T cells, Z86 efficiently inhibited the Topflash activity in both cancer cell lines in a dose-dependent manner after 24 h of treatment (Figure 2a). To check the effects of Z86 on the expression of known target genes of Wnt/*β*-catenin signaling, we monitored mRNA expression of Axin2,^25^ cyclin D1,^7,8^ and protein expression of Axin2, cyclin D1 and Survivin^10,26^ in Z86-treated SW480 and HCT116 cells, as well as *Wnt1-*transfected HEK293T cells. The results showed that, consistent with the Topflash reporter assays, Z86 reduced the expression of these Wnt signaling target genes at both the mRNA and protein levels in a dose- and time-dependent manner in these cells (Figures 2b and c),

To further confirm the inhibitory activity of Z86 on Wnt signaling, we tested its activity in *Xenopus* embryos to inhibit secondary axis induced by Wnt8, a classical model to study Wnt activity. In this assay, Z86 treatment clearly reduced the ratio of injected embryos with secondary axis (Figure 2d), suggesting the inhibition of Wnt signaling by Z86.

### Z86 promotes phosphorylation and degradation of *β*-catenin

**Figure 3:**
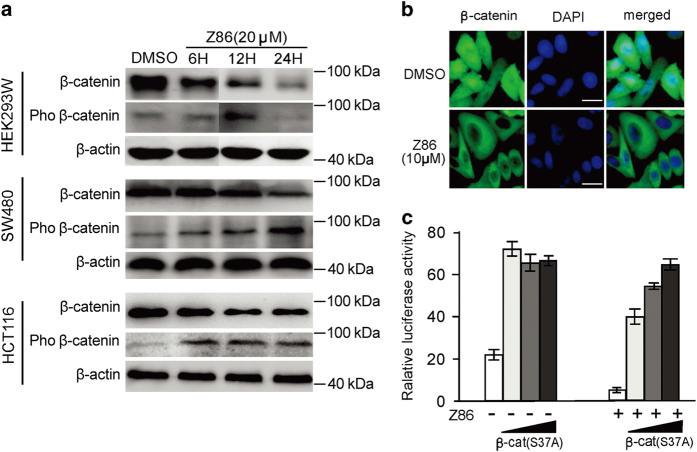
Z86 promotes the phosphorylation and degradation of *β*-catenin. (**a**) Z86 promotes phosphorylation and the subsequent degradation of *β*-catenin in *Wnt3a* stably transfected HEK293W cells, as well as in CRC cells (SW480 and HCT116). Cells were treated with Z86 for different times indicated. The cell extracts were harvested and assayed by western blotting for the expressions of phosphorylated *β*-catenin (Ser33, Ser37 and Thr41) and the total *β*-catenin. *β*-Actin was used as the loading control. The blots show representative data derived from three experiments. (**b**) Cellular distribution and amount of *β*-catenin in SW480 cells. After incubation with Z86 for 12 h, cells were subjected to immunofluorescent staining for *β*-catenin. The microscopy images are representative examples from one of three independent experiments. *β*-Catenin (green) was observed present in the cytosol and nucleus fractions, whereas nucleic acid dyed with 6'-diamidino-2-phenylindole (DAPI; blue) in the nucleus. Scale bars=20 *μ*m. (**c**) The Wnt signaling (ST-Luc) activity in HCT116 cells, transfected with increasing amount of mutant *β*-catenin (S37A), with or without Z86 treatment, was measured using dual-luciferase reporter assay.

*β*-Catenin is an important component of the canonical Wnt signaling pathway that is phosphorylated by the destruction complex and then ubiquitinated and degraded through the proteasome pathway. Elevated *β*-catenin has been frequently reported in colorectal tumors because of the aberrant activation of Wnt signaling. To investigate the molecular mechanism of Z86 that inhibits Wnt signaling pathway, the protein levels of total *β*-catenin and phosphorylated *β*-catenin in Z86-treated Wnt-stimulated HEK293 cells as well as in colon cancer cell lines were checked. In HEK293 cells with *Wnt3a* stably transfected (HEK293W), Z86 treatment reduced *β*-catenin protein levels time dependently, with the phosphorylation of *β*-catenin (Ser33, Ser37 and Thr41) increased markedly. Consistently, similar effects were observed in SW480 and HCT116 CRC cells (Figure 3a). Accumulation and nucleus translocation of *β*-catenin is the hallmark of activation of Wnt signaling and has been extensively observed in CRC. By immunofluorescence assays, we observed that *β*-catenin protein levels were clearly reduced in the nucleus of SW480 cells treated with Z86 (Figure 3b). When a mutant *β*-catenin (S37A), which can no longer be phosphorylated by GSK3*β* and becomes stabilized,^27–29^ was transfected into HCT116 cells, the inhibition of Wnt signaling by Z86 was efficiently rescued, in a dose-dependent manner, suggesting that Z86 likely works upstream of *β*-catenin (Figure 3c).

### Z86 inhibits Wnt signaling through activation of GSK3*β*

**Figure 4:**
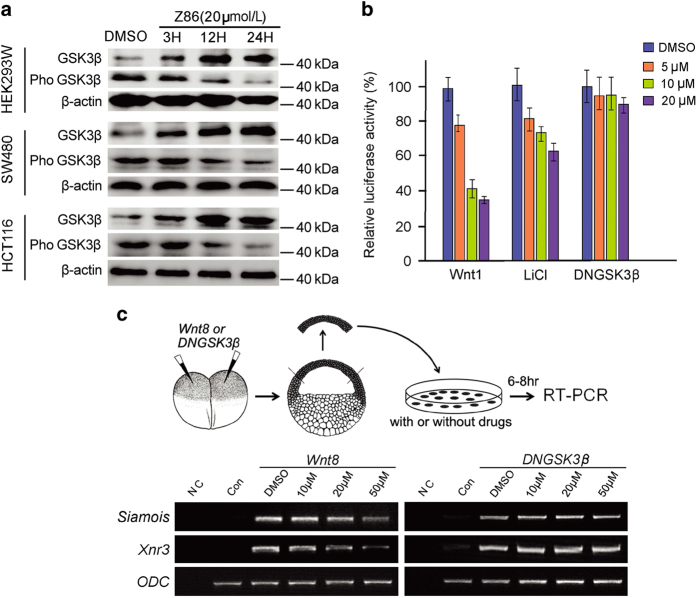
Z86 inhibits Wnt signaling through suppression of GSK3*β* phosphorylation. (**a**) Z86 reduces the phosphorylation of GSK3*β* (Ser9) in both *Wnt3a* stably transfected HEK293W cells and CRC cells. Cells were incubated with 20 *μ*M of Z86, and cell extracts were subjected to western blot analysis to detect phosphorylated GSK3*β* (Ser9) and the total GSK3*β*. *β*-Actin was used as the loading control. (**b**) HEK293T cells were transfected with luciferase reporters (Topflash and *Renilla*) together with Wnt1 (64 ng per well), DNGSK3*β* (30 ng per well) in 96-well plates, or incubated 20 mM of LiCl respectively. After transfection or treatment for 3 h, cells were treated with Z86 at indicated dosage for additional 24 h and luciferase activities were then measured. (**c**, upper panel) Schematic drawing showing the procedure of *Xenopus* animal cap RT-PCR assays. (**c**, lower panel) Z86 inhibits Wnt8, but not DNGSK3*β*-induced expression of *Siamois* and *Xnr3* in *Xenopus* animal cap cells. The experiment was repeated three times and representative results are shown. Amplification of ornithine decarboxylase (ODC) confirmed RNA integrity. NC, negative control with the reverse transcriptase omitted in the RT reaction; Con, control.

GSK3*β* is the central component in the multiprotein destruction complex consisting of GSK3*β*/Axin/APC/casein kinase 1 (CK1) that controls the phosphorylation and degradation of *β*-catenin, and functions as a negative regulator of Wnt signaling pathway.^30–32^ Therefore, we further investigated whether GSK3*β* is involved in Z86-promoted *β*-catenin phosphorylation and degradation in colon cancer cell lines. Interestingly, phosphorylated GSK3*β* (Ser9), the inactive status, significantly reduced upon Z86 treatment in HEK293W cells as well as in HCT116 and SW480 cells, with slight increase of the total level of GSK3*β* protein (Figure 4a). In contrast, LiCl, an inhibitor of GSK3*β*, increased the phosphorylation of GSK3*β* (Ser9) in our study (data not shown), consistent with previous study.^33^ We then tested whether LiCl or dominant-negative kinase-inactive GSK3*β* (DNGSK3*β*)^30^ could rescue the inhibitory activity of Z86 on the Wnt signaling. HEK293T cells were transiently transfected with *Wnt1* and *DNGSK3β* or pretreated with LiCl, respectively, before the cells were treated with Z86, and then Wnt reporter activities were monitored. Z86 efficiently antagonized Wnt1-induced activation of the canonical Wnt signaling but exhibited much weaker inhibitory activity on the Topflash activity stimulated by LiCl and, furthermore, lost the inhibitory activity completely when the Wnt signaling was stimulated with DNGSK3*β* (Figure 4b).

We also used *Xenopus* animal cap assay to investigate the level at which Z86 inhibits Wnt signaling. In this assay, with increasing doses, Z86 gradually inhibited the expression of the Wnt target genes, *siamois*^34,35^ and *xnr3*,^36^ induced by *Wnt8* injection. On the contrary, when DNGSK3*β* was used to induce the expression of *siamois* and *xnr3*, no clear inhibition could be observed by Z86 at the doses tested (Figure 4c). These data suggest that Z86 works at the level of GSK3*β* or upstream that leads to the activation of the destruction complex and promotes the phosphorylation of *β*-catenin.

### Z86 inhibits the growth of colon cancer cells with G1-phase arrest of cell cycle

**Figure 5:**
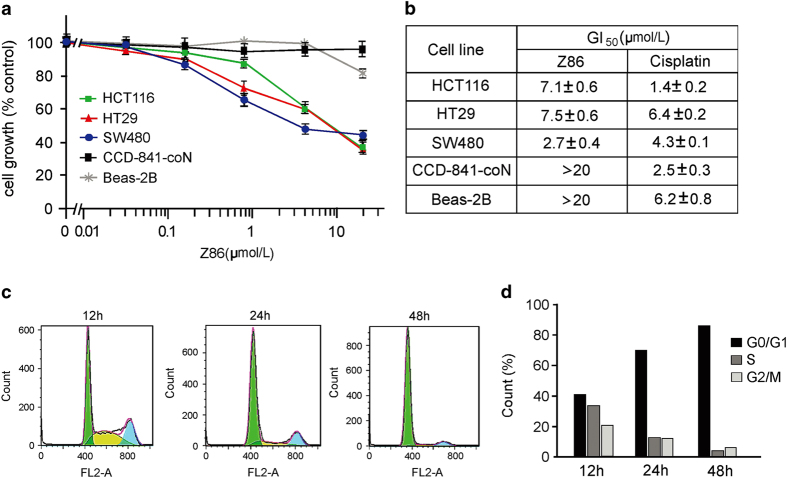
Z86 inhibits the growth of colon cancer cells with G1-phase arrest. (**a** and **b**) Growth inhibition of Z86 on various cell lines, including CRC cell lines (SW480, HCT116 and HT29), normal colonic epithelial cell line CCD-841-CoN and human normal bronchus epithelial cell line Beas-2B, determined by MTS assays with cisplatin used as a control. Cells were treated with Z86 up to 72 h at various concentrations. All samples were done in triplicates and the median growth inhibition concentration (GI_50_) values of Z86 and cisplatin toward the cell lines are presented as mean±S.D. (**c** and **d**) Representative histograms depicting cell cycle distribution as analyzed by flow cytometry in HCT116 cells treated with 20 *μ*M of Z86 for 12, 24 and 48 h respectively. Cell counts of different phases were quantified.

We evaluated the growth inhibition effects of Z86 on human CRC cells with MTS (3-(4,5-dimethylthiazol-2-yl)-5-(3-carboxymethoxyphenyl)-2-(4-sulfophenyl)-2H-tetrazolium) assay. As shown in Figures 5a and b, Z86 selectively inhibited the growth of colon cancer cells with constitutively activated Wnt signaling, including SW480, HCT116 and HT29, whereas it showed relatively low toxicity on normal epithelial cell lines, including CCD-841-CoN (human normal colonic epithelial cell line) and Beas-2B (human normal bronchus epithelial cell line). In contrast, cisplatin, a commonly used chemotherapy drug, showed general cytotoxicity against all the cell lines tested.

Cyclin D1 is a key regulator of cell cycle and has a direct effect on cell proliferation. The expression of cyclin D1 is strongly dependent on *β*-catenin/TCF and the dominant-negative TCF causes cells to arrest in the G1 phase of the cell cycle. Previous works provided evidences that mutant *β*-catenin produces high levels of cyclin D1 messenger RNA and protein constitutively in colon cancer cells.^8^ Z86 reduced the expression of cyclin D1 at both the mRNA and protein levels in a dose- and time-dependent manner in HCT116 and other colon cells (Figures 2b and c). Consistent with these observations, HCT116 cells treated with Z86 were also arrested efficiently at the G1 phase (Figures 5c and d).

### Z86 inhibits tumor growth through suppression of GSK3*β* phosphorylation in colorectal tumor xenografts

**Figure 6:**
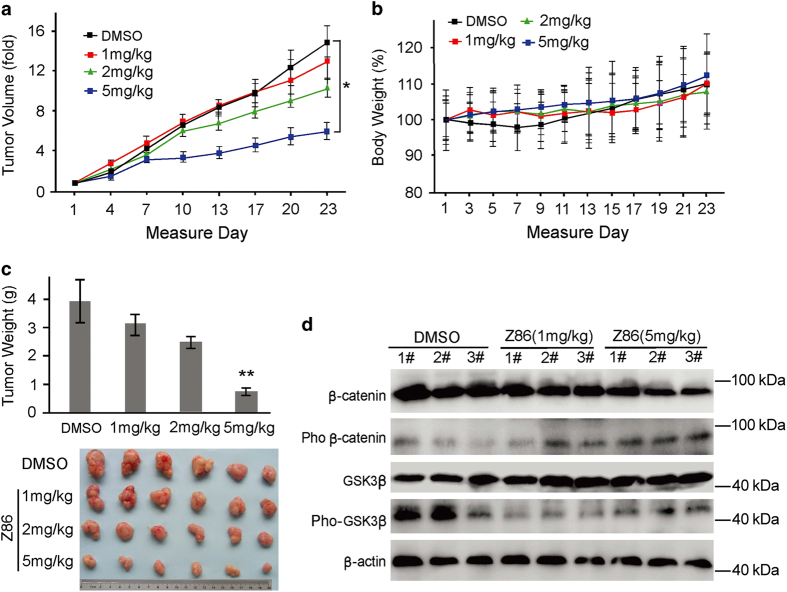
Z86 inhibits tumor growth of HCT116 xenografts through suppression of GSK3*β* phosphorylation *in vivo*. Mice were injected with 5×10^6^ of HCT116 cells. One week later, tumor-bearing mice were treated intraperitoneally with either vehicle or Z86 (1, 2 and 5 mg/kg respectively) daily for 23 days. (**a** and **b**) Tumor growth and body weight were monitored during the administration. (**c**) Tumors were removed from mice and tumor weight was measured on day 23. Data represent the mean±S.D., *P<0.05, **P<0.01, relative to vehicle control. (**d**) The expression of *β*-catenin and phosphorylated *β*-catenin (Ser33, Ser37 and Thr41) as well as GSK3*β* and phosphorylated GSK3*β* (Ser9) in tumor tissues was determined by western blot analysis.

To assess the *in vivo* antitumor effect of Z86, human colon cancer HCT116 xenografts were established in nude mice. HCT116 cells were injected subcutaneously into the flanks of nude mice to initiate tumor formation. Tumor-bearing mice were randomly divided into four groups and intraperitoneally injected with three dosages of Z86 (1, 2 and 5 mg/kg) and vehicle daily for 21 days. The results showed that 5 mg/kg of Z86 dramatically reduced the growth of HCT116 xenografts in both tumor volume (Figure 6a) and tumor weight (Figure 6b) compared with the vehicle-treated group. Meanwhile, Z86 treatment did not affect body weight as no difference in body weight was observed between control and Z86-treated animals (Figure 6c). These data demonstrated that Z86 could inhibit effectively the tumor growth *in vivo* without clear toxicity.

Next, we checked the protein levels and phosphorylation status of *β*-catenin and GSK3*β* in tumor tissues. As expected, Z86 treatment reduced the phosphorylation of GSK3*β* (Ser9) and increased the levels of phospho-*β*-catenin (Ser33/Ser37/Thr41) in the tumor tissues compared with the vehicle-treated group. The total level of GSK3*β* increased slightly and that of *β*-catenin decreased as compared with the control groups (Figure 6d). These findings are consistent with the data shown in the culture cells, demonstrating that targeting GSK3*β* kinase to promote *β*-catenin degradation contributes to the antitumor activity of Z86 both *in vitro* and *in vivo*.

## Discussion

Altered function and expression of Wnt/*β*-catenin signaling components that result in *β*-catenin stabilization, such as mutations of APC, Axin or *β*-catenin itself, are known to contribute to a wide range of cancers, particularly CRC.^6,37–41^ In the present study, we described the discovery of a novel *β*-carboline structure-type compound Z86 that interfered with Wnt/*β*-catenin pathway by overactivation of GSK3*β*, promoted *β*-catenin degradation and finally exhibited efficient cell growth inhibition in colon cancer cells both *in vitro* and *in vivo*.

It is well established that the constitutive Wnt signaling is essential for the CRC cell proliferation, and that the suppression of the Wnt signaling pathway can result in the inhibition of cell growth. Consistently, our data demonstrated that Z86 exhibited growth inhibitory effect on CRC cells. Importantly, there was no growth reduction observed in Z86-treated normal colonic epithelial cell line CCD-841-CoN cells that lack aberrantly activated endogenous canonical Wnt signaling, indicating the selective growth inhibitory effect between cancer cells and normal cells was attributed to the inhibition of Wnt signaling (Figures 5a and b). Thus, we expect that Z86 could be developed as a potential anticancer agent targeting overactivated Wnt signaling with minor side effects on normal cells. Many target genes of Wnt/*β*-catenin pathway, such as c-MYC and cyclin D1, play critical roles in tumorigenesis. Our findings showed that Z86 downregulated the expression of cyclin D1 and resulted in G1-phase arrest in HCT116 cells, suggesting that cell growth inhibition of Z86 is mediated by the modulation of cell cycle distribution (Figures 5c and d). To further assess the anticancer effects of Z86, we extended our studies to *in vivo* human CRC xenografts by implanting HCT116 tumor cells in nude mice. The results showed that Z86 treatment resulted in dramatic decrease in tumor growth, with no detectable side effect in the mice by monitoring the body weight (Figure 6).

In CRCs where Wnt signaling is activated by mutated APC or *β*-catenin, it seems that the ideal antagonist of the pathway would be the one that works in the nucleus. Accordingly, small molecules first identified as Wnt inhibitors targeted exactly the level of transcriptional complexes and inhibited CRC cell proliferation.^15–20^ However, there are recently some experimental results showing that, at least in some cases, targeting the upstream components of the Wnt signaling pathway can also play a role. A couple of small molecules have been reported targeting the GSK3*β*/Axin/APC/CK1 complex, the so-called *β*-catenin destruction complex. Representative small molecules, such as XAV939,^19^ JW74,^20^ IWR,^21^ JW55,^42^ and G007-LK,^22^ stabilized Axin and exhibited promising inhibitory activity on Wnt signaling in APC or *β*-catenin-mutant CRC models. As the central component of the destruction complex, GSK3*β* is a cytoplasmic serine/threonine protein kinase that serves as negative regulator and plays a key role in regulating Wnt signaling through phosphorylation of *β*-catenin.^43–46^ Therefore, GSK3*β* is recognized as an attractive target for the development of new antitumor drugs.^47^ In our study, we found that Z86 effectively inhibited GSK3*β* phosphorylation, leading to overactivation of GSK3*β*, promoted degradation of *β*-catenin and inhibition of Wnt signaling. Treatment with LiCl or transfection with DNGSK3*β* rescued effectively the intracellular *β*-catenin/TCF transcriptional activity inhibited by Z86, indicating that Z86 inhibited Wnt signaling through activation of GSK3*β* in colon cancer cells (Figure 4b). Z86 inhibited efficiently colon cancer cell growth both *in vitro* and *in vivo* (Figures 5 and 6), supporting further antagonists targeting the upstream components of Wnt signaling work in colon cancer suppression.

It should be noted that previous work suggested that in the *β*-catenin mutation sites, the serines at positions 37 and 45 are likely important phosphorylation sites, as the substitution of Ser37 and Ser45 markedly reduced the phosphorylation of *β*-catenin in many cancer cell lines.^38^ Mutation of Ser45 could block the phosphorylation at positions Ser33, Ser37 and Thr41.^29^ However, in HCT116 cells that harbor a single-point mutation at Ser45 in one *β*-catenin allele, Z86 also works effectively to promote the phosphorylation and degradation of *β*-catenin and subsequent inhibition of Wnt signaling. This is consistent with the finding that phosphorylation of *β*-catenin at Ser33, Ser37 or Thr41 can occur in the absence of phosphorylation at Ser45 in colon cancer cells.^48^ The small-molecule JW74, which works through stabilization of Axin, was also reported attenuating Wnt signaling in HCT116 cells.^20^

In conclusion, our data indicated that Z86 effectively inhibits colon cancer cell proliferation *in vitro* and *in vivo* by blocking the Wnt/*β*-catenin pathway. We showed that reduced phosphorylation of GSK3*β* is involved in the inhibitory activity of Z86 on Wnt signaling, and the direct targets of Z86 are under investigation. Our findings provide a novel chemotype of antagonists of the canonical Wnt signaling and highlight a promising candidate for further CRC therapeutics development.

## Materials and Methods

### Reagents

RPMI-1640 medium, Dulbecco’s modified Eagle’s medium (DMEM), fetal bovine serum (FBS), antibiotics–antimycotics solution and trypsin–EDTA were purchased from HyClone (Logan, UT, USA). Complete protease inhibitor cocktail was purchased from Roche Applied Science (Indianapolis, IN, USA). Mouse monoclonal anti-*β*-catenin antibodies were purchased from BD Biosciences (San Jose, CA, USA). Other reagents unless otherwise indicated were purchased from Sigma-Aldrich (St. Louis, MO, USA). Lipofectamine 2000 was from Invitrogen (Camarillo, CA, USA). Dual-luciferase reporter assay system was purchased from Promega (Madison, WI, USA). Tumor necrosis factor-*α* (TNF*α*) was from PeproTech (Rocky Hill, NJ, USA) and LiCl was purchased from Sigma-Aldrich. QuantiTect SYBR Green PCR Kit was obtained from QIAGEN (Hilden, Germany).

### Plasmids

The pF9A CMV hRluc-neoFIexi reporter plasmid (*Renilla*) was from Promega. The SuperTopflash, pCS-mWnt1, human *β*-catenin and *β*-catenin (S37A) plasmids were gifted by Dr. Wei Wu (Tsinghua University, Beijing, China). *Xenopus* Wnt8 and dominant-negative GSK3*β* (K85R) expression vectors were gifted by Dr. Aaron Zorn (Cincinnati Children’s Hospital Medical Center, Cincinnati, OH, USA). NF-*κ*B-luc was from Beyotime Biotechnology (Shanghai, China).

### Cell culture

Colon cancer cell lines (SW480, HT-29 and HCT116), human normal bronchus epithelial cell line (Beas-2B) and HEK293T were purchased from ATCC (Manassas, VA, USA). Cells were cultured in medium (DMEM for SW480, HCT116, Beas-2B and HEK293T cells, RPMI-1640 medium for HT-29), supplemented with 10% FBS and 1% antibiotics (100 units/ml penicillin, 100 *μ*g/ml streptomycin) (Bioind, Kibbutz Beit Haemek, Israel). Normal colonic epithelial cell line (CCD-841-CoN) was kindly gifted by Professor Lin Li (Institute of Biochemistry and Cell Biology, Chinese Academy of Sciences, Shanghai, China) and was maintained according to the recommendations of ATCC. All the cells were incubated at 37 °C, 5% CO_2_ in a humidified atmosphere, and were passaged once or twice a week.

### Screening of Wnt signaling inhibitors

To identify novel small-molecule inhibitors of the Wnt signaling pathway, cell line with stable expression of Wnt3a and dual luciferase reporters (SuperTopflash and *Renilla*) was generated in HEK293 (named HEK293W). This cell-based screening strategy was sensitive to Wnt modulators as previously reported.^17,49^ Briefly, HEK293W cells were maintained in DMEM medium containing 200 *μ*g/ml G418 and 100 *μ*g/ml Hygromycin B (Sigma-Aldrich), respectively. Luciferase assay was carried out 24 h after compounds addition using SteadyGlo reagent (Promega). For the primary screen, compounds with the inhibition rate of >70% with concentration at 20 *μ*M in 0.1% dimethylsulfoxide (DMSO) were identified as positive hits. Secondary screenings were carried out further with successive concentrations to confirm inhibitory activity with IC_50_ of the positive hits.

### Reporter activity assay

HEK293T, SW480 and HCT116 cells were seeded in 96-well plates containing 100 *μ*l DMEM and 10% FBS. Cells were incubated at 37 °C and 5% CO_2_ incubator for 12 h. Each well of SW480 and HCT116 cells were transfected with 90 ng of plasmids in total, including 80 ng of SuperTopflash (*β*-catenin/TCF reporter plasmids) and 10 ng of pF9A CMV hRluc-neoFIexi using Lipofectamine 2000 according to the manufacturer’s instructions. Each well of HEK293T cells was transfected with 200 ng of plasmids in total, including 80 ng of Topflash, 10 ng of *Renilla* and 10 ng of *β*-catenin or 64 ng of Wnt1 and other plasmids as indicated. At 3 h after transfection, cells were incubated with test compounds for 24 h. Cells were lysed and luciferase activity was measured with Dual-Luciferase Reporter Assay System (Promega). Transfection efficiency was normalized by the values of *Renilla* luciferase activity as previously described.^17^ All experiments were done independently in triplicate and the results reported are the means and S.D.s.

### Reverse transcription-PCR (RT-PCR) and quantitative real-time PCR

Total RNAs were extracted from cultured cells with TRIzol according to the manufacturer’s instructions (Invitrogen). cDNA synthesis, reverse transcription and PCR were performed as previously described.^17^ For quantitative, all gene transcripts were done by real-time PCR and two-step RT-PCR using SYBR Green I (QIAGEN). The values of Axin2 and cyclin D1 were shown against the value of *β*-actin that was used as the control. Primer sequences are provided in Supplementary Table S1.

### Western blotting and immunofluorescence analyses

Cells seeded in six-well plates were subjected to different treatments and the immunofluorescence staining of *β*-catenin in SW480 cells was performed as previously described.^49^ Western blots were performed as described previously.^17^ Primary antibodies used include: phospho-*β*-catenin (Ser33/37/Thr41), phospho-GSK3*β* (Ser9), GSK3*β*, Axin2 (Cell Signaling, Danvers, MA, USA), Survivin, *β*-catenin (BD Biosciences), cyclin D1 (Santa Cruz, Dallas, TX, USA) and *β*-actin (Sigma-Aldrich).

### MTS assay and determination of GI_50_

MTS assays were performed as previously described.^17^ GI_50_ values, the concentrations for 50% cell growth inhibition, were determined by nonlinear regression analysis using Table curve software (Systat Software, San Jose, CA, USA).

### Flow cytometry analyses

HCT116 cells (5×10^5^ cells/dish in 60 mm dishes) were incubated with test samples for 12, 24 and 48 h, respectively. All adherent cells were collected and washed twice with PBS. Cells were fixed with 70% ethanol overnight. Fixed cells were washed with PBS, and then stained with 50 *μ*g/ml propidium iodide (PI) solution containing 50 *μ*g/ml RNase A for 30 min in dark at room temperature. Fluorescence intensity was analyzed by FACSCalibur flow cytometer (BD Biosciences). The percentages of the distributions in distinct phases of cell cycle were determined using ModFIT LT V2.0 software (Verity Software, Topsham, ME, USA).

### *Xenopus* studies

Adult *Xenopus laevis* frogs were obtained from Nasco (Fort Atkinson, WI, USA). The *in vitro fertilization*, embryo culture, staging and microinjection were carried out as previously described.^50^ For microinjection, capped Wnt8 and DNGSK3*β* mRNAs were synthesized using SP6 mMessagemMachine kit (Ambion, Carlsbad, CA, USA) with their expression vectors linearized with *Not*I as templates. For secondary axis induction, 5 pg Wnt8 mRNA was injected into the ventral blastomeres of four-cell-stage embryos. For animal cap RT-PCR assays, 5 pg Wnt8 or 100 pg DNGSK3*β* were injected into the dorsal blastomeres of two-cell-stage embryos, and animal caps were excised from late blastula-stage embryos and then treated with test compounds for 6–8 h or not before being harvested. Primer sequences are provided in Supplementary Table S2.

### Mouse *in vivo* studies

Male nu/nu mice (3 weeks old) were purchased from Vital River Laboratory Animal Technology (Beijing, China) and acclimated for 4 days. HCT116 cells (5×10^6^) in 200 *μ*l of PBS were implanted into the right flank of each nude mouse. Tumor size was measured in two dimensions with calipers and calculated using the formula (*L*×*W*^2^)/2, where *L* is length and *W* is width. After the tumor size reached ∼80 mm^3^, which occurred 7 days after cell injection, the mice were randomly assigned into three Z86 treatment groups with dose of 1, 2 and 5 mg/kg and a vehicle control group. Z86 in 100 *μ*l PBS (with 1% DMSO and 5% Tween-80) was injected intraperitoneally into each mouse daily for 21 days. Mice were intraperitoneally injected with 100 *μ*l PBS (with 1% DMSO and 5% Tween-80) as a vehicle control daily for 21 days. The mice body weight and tumor size was measured every 2 days and every 3 days, respectively. Tumors were removed from mice 23 days after treatment. Tumor weight was measured and tumor tissue was flash frozen at −80 °C for subsequent western blotting assay.

### Statistical analyses

Data were presented as means and S.D.s for the indicated number of independently performed experiments. Figure data are shown as one representative of at least two independent experiments. Nonlinear regression analysis for calculation of GI_50_ or IC_50_ values was performed by Table curve program. Statistical significance was analyzed by Student’s *t*-test or one-way ANOVA (SigmaStat 3.1, Systat software Inc., San Jose, CA, USA). The difference was considered to be statistically significant when *P*<0.05.

## Abbreviations

APC: adenomatous polyposis coli
CK1: casein kinase 1
c-MYC: v-myc avian myelocytomatosis viral oncogene homolog
CRC: colorectal cancer
DMSO: dimethylsulfoxide
DNGSK3*β*: dominant-negative form of GSK3*β*
Dsh: Dishevelled family member
GI_50_: 50% growth inhibition (72 h of treatment)
GSK3*β*: glycogen synthase kinase-3*β*
IC_50_: median inhibitory concentration
LEF: lymphoid enhancer factor
MTS: 3-(4,5-dimethylthiazol-2-yl)-5-(3- carboxymethoxyphenyl)-2-(4-sulfophenyl)-2H-tetrazolium
NF-*κ*B: nuclear factor-*κ*B
PI: propidium iodide
RT-PCR: reverse transcription-PCR
TCF: T-cell factor
TNF*α*: tumor necrosis factor-*α*
Z86: isopropyl 9-ethyl-1-(naphthalen-1-yl)-9H-pyrido[3,4-b]-indole-3-carboxylate.
