# Supplementary material for: Novel β-carbolines against colorectal cancer cell growth via inhibition of Wnt/β-catenin signaling
Source: Cell Death Discov. 2015 Oct 5;1:15033–. doi: 10.1038/cddiscovery.2015.33 (PMC4979417; doi:10.1038/cddiscovery.2015.33)

**Supplementary Information**

**Chemicals**

**Typical procedure for synthesis and information of Z86 and its analogs.**

1. 1-naphthaldehyde (10 mmol) was added into a solution of L-tryptophan ester (10 mmol) in 30 mL dry CH_2_Cl_2_ in the presence of 4 Å molecular sieves and stirred at r.t for 48 h, then TFA(10 mmol) was added into the solution and stirred for 24 h. cooled to 0 °C. After checking by TLC, it was quenched with saturated NaCl. The solution was extracted with ethyl acetate and the combined organic solution was dried over anhydrous Na_2_SO_4_. The solvents were removed under reduced pressure to give a pale yellow powder.
2. Tetrahydro-β-carboline(0.25 mmol) was dissolved in 10 mL CH_2_Cl_2_, then DDQ (0.5 mmol)was added slowly. After reaction at r.t for 30 min, the mixture was washed with water and and dried under vacuum to give a pale yellow powder.
3. K_2_CO_3_ powder（0.5 mmol）was suspended in anhydrous DMF under a nitrogen atmosphere.β-carboline (0.25 mmol) was added following by bromoethane (0.75 mmol) and the mixture was stirred for 12 h at r.t, and then poured into ice-water. The precipitate was collected by filtration, washed with water and dried under vacuum to give a white power.

**Z64** (Methyl 9-ethyl-1-(naphthalen-1-yl)-9H-pyrido[3,4-b]indole-3-carboxylate):

MS-ESI, *m/z* 381 [M+H]^+^. ^1^H NMR (500 MHz, CDCl_3_) *δ*0.76 (3H, t, *J* = 7.0 Hz), 3.43-3.72 (2H, m), 4.03 (3H, s), 7.31 (2H, m), 7.40 (2H, m), 7.49 (1H, t, *J* = 7.5 Hz), 7.63 (2H, m), 7.72 (1H, d, *J* = 6.8 Hz), 7.95 (1H, d, *J* = 8.2 Hz), 8.02 (1H, d, *J* = 8.2 Hz), 8.32 (1H, d, *J* = 7.9 Hz), 9.04 (1H, s). ^13^C NMR (100 MHz, CDCl_3_) *δ* 14.2, 39.1, 52.7, 110.2, 117.2, 120.8, 121.8, 121.9, 125.3, 125.7, 126.2, 126.7, 127.4, 128.1, 128.8, 129.1, 129.7, 132.6, 133.4, 136.5, 137.1, 141.8, 142.4, 166.9.

**Z80** (Ethyl 9-ethyl-1-(naphthalen-1-yl)-9H-pyrido[3,4-b]indole-3-carboxylate):

MS-ESI, *m/z* 395 [M+H]^+^. ^1^H NMR (400 MHz, CDCl_3_) *δ*0.75 (3H, t, *J* = 7.1 Hz), 1.46 (3H, t, *J* = 7.1 Hz), 3.46 (1H, m), 3.69 (1H, m), 4.54 (2H, m), 7.29-7.39 (4H, m), 7.49 (1H, t, *J* = 7.1 Hz), 7.63 (2H, m), 7.71 (1H, d, *J* = 6.9 Hz), 7.95 (1H, d, *J* = 8.2 Hz), 8.00 (1H, d, *J* = 8.2 Hz), 8.31 (1H, d, *J* = 7.9 Hz), 8.99 (1H, s). ^13^C NMR (100 MHz, CDCl_3_) *δ* 14.2, 14.5, 39.0, 61.5, 110.2, 117.0, 120.7, 121.7, 121.8, 125.3, 125.8, 126.1, 126.6, 127.4, 128.3, 128.8, 129.1, 129.7, 132.6, 133.4, 136.4, 136.5, 137.4, 141.8, 142.4, 166.2.

**Z83** (Isopropyl 1-(naphthalen-1-yl)-9H-pyrido[3,4-b]indole-3-carboxylate):

MS-ESI, *m/z* 381 [M+H]^+^. ^1^H NMR (400 MHz, CDCl_3_) *δ* 1.45 (6H, d, *J* = 6.3 Hz), 5.38 (1H, m), 7.37 (3H, m), 7.50-7.57 (3H, m), 7.73 (2H, m), 7.94 (2H, m), 8.24 (1H, d, *J* = 7.9 Hz), 8.34 (1H, s), 8.89 (1H, s). ^13^C NMR (100 MHz, CDCl_3_) *δ*22.0, 68.9, 111.9, 116.8, 120.9, 121.9, 122.0, 125.5, 126.1, 126.7, 128.1, 128.5, 128.8, 129.0, 129.5, 131.1, 133.9, 134.5, 136.2, 138.8, 140.4, 142.8, 165.6.

**Z86** (Isopropyl 9-ethyl-1-(naphthalen-1-yl)-9H-pyrido[3,4-b]indole-3-carboxylate):

MS-ESI, *m/z* 409 [M+H]^+^. ^1^H NMR (400 MHz, CDCl_3_) *δ*0.75 (3H, t, *J* = 7.1 Hz), 1.46 (6H, d, *J* = 6.3 Hz), 3.45 (1H, m), 3.69 (1H, m), 5.39 (1H, m), 7.29 (1H, d, *J* = 8.2 Hz), 7.39 (2H, m), 7.48 (1H, t, *J* = 6.9 Hz), 7.63 (2H, m), 7.71 (1H, d, *J* = 6.4 Hz), 7.94 (1H, d, *J* = 8.2 Hz), 8.00 (1H, d, *J* = 8.1 Hz), 8.31 (1H, d, *J* = 7.9 Hz), 8.92 (1H, s). ^13^C NMR (100 MHz, CDCl_3_) *δ* 14.2, 22.0, 39.0, 68.8, 110.3, 116.7, 120.6, 121.7, 121.8, 125.2, 125.8, 126.1, 126.6, 127.4, 128.3, 128.7, 129.1, 129.6, 132.6, 133.3, 136.3, 136.6, 137.7, 141.8, 142.6, 165.3.


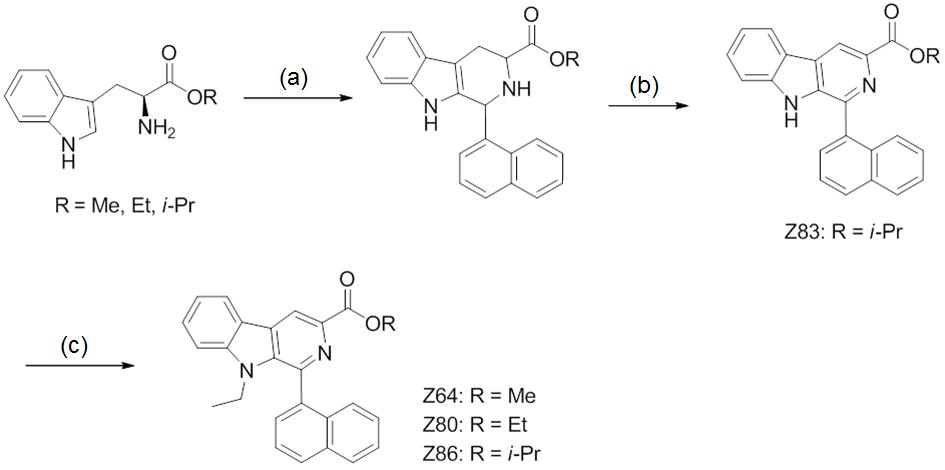


**Figure S1.** Synthetic schemes of Z86 and analogs. (**a**) aldehyde, CH_2_Cl_2_, molecular sieve, r.t, 48 h, then TFA, 24 h; (**b**) DDQ, CH_2_Cl_2_, r.t, 0.5 h; (**c**) CH_3_CH_2_Br, K_2_CO_3_, DMF, r.t, 12 h.


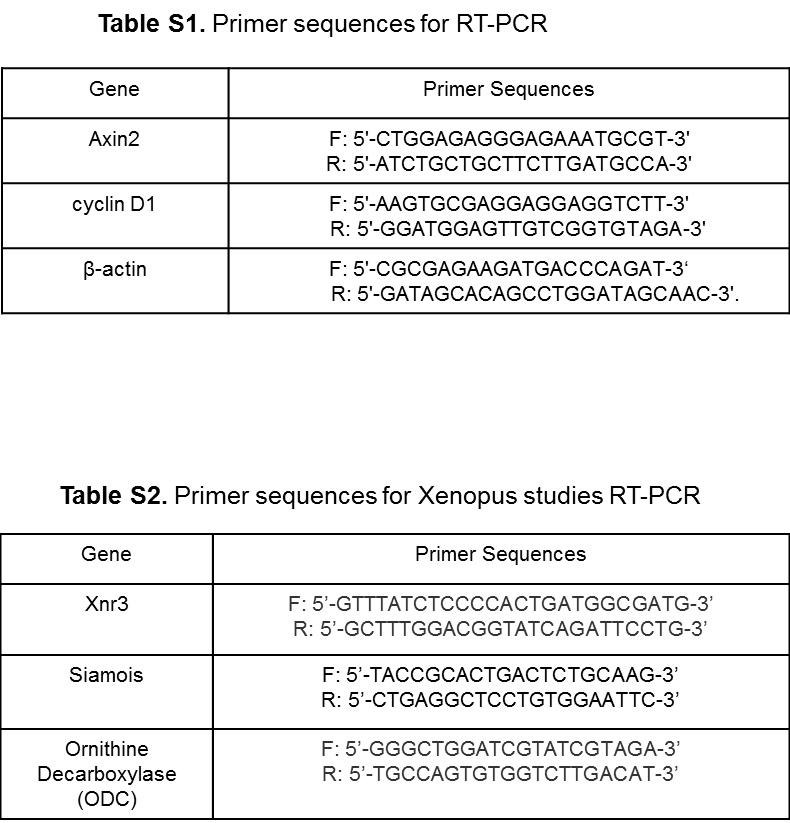

Supplement: Supplementary Information [file cddiscovery201533-s1.doc]
